# Applicability of an ultrasound scoring system for predicting hemorrhage during cesarean section in patients with placenta accreta spectrum disorders

**DOI:** 10.3389/fmed.2026.1814921

**Published:** 2026-09-08

**Authors:** Wenying Liu, Ge Zhang, Bocheng Liang, Lijun Song, Yanjie Wang, Peixin Lin, Hong Yin

**Affiliations:** 1 Department of Ultrasound, Key Laboratory of Maternal & Fetal Medicine of National Health Commission of China, Shandong Maternal and Child Health Hospital, Jinan, China; 2 Department of Ultrasound, Shenzhen Maternity and Child Healthcare Hospital, Shenzhen, China

**Keywords:** cesarean section, hemorrhage, placenta accreta spectrum disorders, predictive value, ultrasound scoring system

## Abstract

**Objective:**

To investigate the value of an ultrasound scoring system for predicting hemorrhage during cesarean section in patients with placenta accreta spectrum (PAS) disorders.

**Data and Methods:**

The ultrasound images, ultrasound scores, and clinical data of 63 patients with PAS disorders who were admitted to Maternal and Child Health Care Hospital of Shandong Province between January 2023 and August 2025 were retrospectively analyzed, subjected to univariable and multivariable logistic regression analyses, and evaluated to identify risk factors of intraoperative hemorrhage (blood loss ≥ 1000 mL) during cesarean section. Receiver operating characteristic (ROC) curve analysis was conducted to evaluate the predictive efficacy of the ultrasound scoring system for massive hemorrhage during cesarean section in patients with PAS disorders.

**Results:**

Multivariable analysis revealed that the ultrasound score was an independent risk factor for intraoperative hemorrhage during cesarean section in patients with PAS disorders (*P* < 0.05). Among the ultrasound scoring indicators, complete placenta previa and bridging vessels at the placental basal plate were confirmed as independent predictors of intraoperative hemorrhage (*P* < 0.05). ROC curve analysis revealed that in predicting massive intraoperative hemorrhage, the area under the curve (AUC) was 0.884; moreover, at a cutoff value of 7.5, the sensitivity was 80.0%, the specificity was 82.6%, the positive predictive value (PPV) was 88.9%, the negative predictive value (NPV) was 70.4%, the positive likelihood ratio (LR+) was 4.598, and the negative likelihood ratio (LR-) was 0.242.

**Conclusion:**

The ultrasound score is an effective tool for predicting intraoperative hemorrhage during cesarean section in PAS patients. Complete placenta previa, bridging vessels at the placental basal plate, and a score ≥ 7.5 are important risk factors. Accurate identification of these factors is helpful for stratifying patients preoperatively, optimizing surgical preparation, and improving clinical decision-making.

## 1 Introduction

Placenta accreta spectrum (PAS) disorder is a serious complication of pregnancy characterized by abnormal invasion of placental villi into the myometrium, adjacent organs, or uterine wall. During cesarean delivery, patients with PAS carry an extremely high risk of massive intraoperative hemorrhage, a life-threatening complication that may directly lead to multiple maternal organ damage, hemorrhagic shock, and even maternal death (1, 2).

Owing to its convenience and reproducibility and its real-time imaging capabilities, ultrasound has become the preferred method for assessing PAS in pregnant individuals (3–5). Prior to the establishment of a multiparametric ultrasound scoring system, the European Working Group on Abnormally Invasive Placenta (EW-AIP) published internationally standardized sonographic descriptors for PAS in 2016, defining core imaging markers including loss of the retroplacental clear zone, abnormal placental lacunae, disrupted bladder wall continuity, and bridging vessels at the placental basal plate (6). In the United States, PAS diagnosis adheres to the 2018 ACOG consensus, which integrates clinical risk factors—complete placenta previa with prior cesarean deliveries—and characteristic sonographic findings such as abundant placental lacunae, absence of the retroplacental hypoechoic space, myometrial thinning of less than 1 mm, and subplacental hypervascularity (7). Comprehensive guidelines by Alfirevic et al. further validated the diagnostic utility of these standardized signs (8). Despite these consensus frameworks, early PAS diagnosis has largely relied on sonographers’ subjective interpretation, lacking systematic quantitative criteria and resulting in suboptimal diagnostic consistency. Thus, objective quantitative assessment tools are urgently needed to address this limitation (9).

In recent years, multiple quantitative ultrasound scoring systems have been developed for prenatal risk stratification of PAS. A 2024 systematic review by Peng et al. (10) comprehensively compared the discriminatory ability of mainstream scoring tools and confirmed that these existing scoring systems exhibit satisfactory performance for identifying PAS (11–13). The review further mentioned that Watthanasathitnukun et al. reported significant associations of focal exophytic masses, placental bulging and intralacunar trophoblastic vessels with perioperative massive hemorrhage (14). Nevertheless, studies that systematically quantify the independent predictive value of the above ultrasonic markers for intraoperative blood loss remain scarce.

The multiparametric ultrasound scoring system proposed by Chong et al. (15) incorporates core imaging markers recommended by the EW-AIP alongside several newly added critical ultrasonic signs, including placental location, placental thickness, cervical venous sinuses, and abnormal cervical morphology. Combined with a history of previous cesarean delivery for quantitative scoring, this system greatly improves the objectivity and reproducibility of prenatal PAS diagnosis (16). Chong et al. also divided PAS disorder into placenta accreta, placenta increta, and placenta percreta using thresholds of 5 and 10 points, reporting that the amount of bleeding in patients with placenta increta and placenta percreta was significantly greater than that in those with placenta accreta, suggesting that higher scores are associated with a greater risk of massive hemorrhage; however, the specific threshold score for massive hemorrhage remained undefined. The ultrasound scoring system assigns the same score to different imaging signs, but the depth of pathological invasion reflected by each sign differs, resulting in considerable differences in the amount of intraoperative blood loss in patients with the same score. Therefore, preoperative identification of specific high-risk sonographic signs is also of significant predictive value.

Currently, studies on the use of prenatal ultrasound signs for predicting the risk of intraoperative hemorrhage during cesarean section in PAS patients have not reached a consensus on sign selection, criteria, or predictive efficacy. In this study, to further clarify the predictive value of specific ultrasound signs and the ultrasound scoring for hemorrhage in PAS patients, the ultrasound scoring system proposed by Chong et al. was used, and the associations between its score and intraoperative blood loss in PAS patients was analyzed, aiming to identify high-risk indicators for massive hemorrhage and establish ultrasound scoring thresholds, thereby improving the utility of the scoring system in clinical decision-making.

## 2 Materials and methods

### 2.1 Research participants

**Figure 1:**
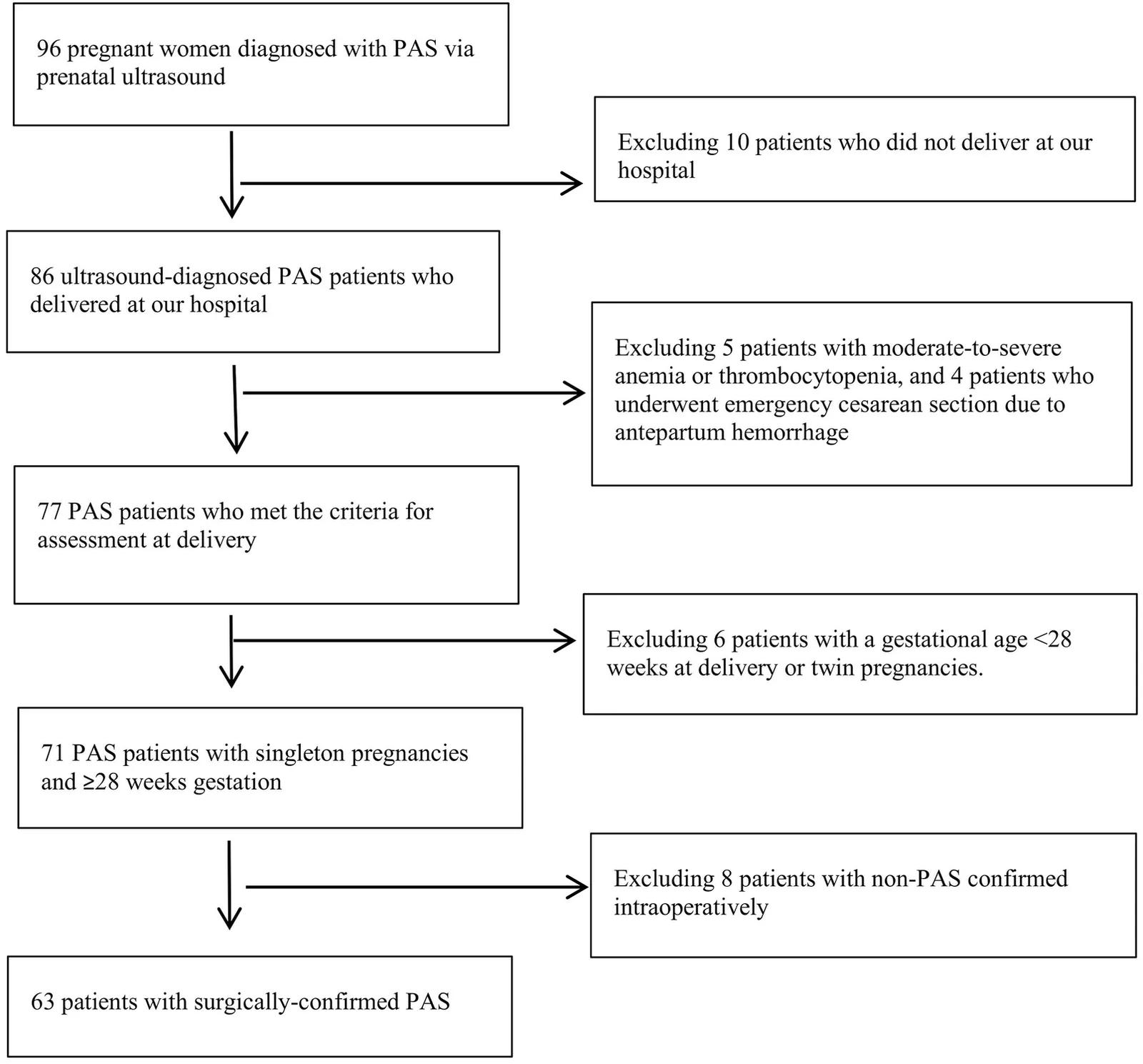
Patient screening flowchart.

A total of 63 patients who were diagnosed with PAS and underwent a cesarean section in the Department of Obstetrics of our hospital between January 2023 and August 2025 were retrospectively included. The inclusion criteria were as follows: (1) pregnancy, a confirmed diagnosis of PAS during cesarean section performed at our hospital and ultrasound scoring completed one week before the operation; (2) All surgeries for patients with PAS at our center are performed by our specialized multidisciplinary team, and all operations are conducted by physicians with associate senior professional titles or higher; (3) fetal gestational age ≥ 28 weeks; and (4) complete ultrasound imaging and clinical data. The exclusion criteria were as follows: (1) other serious complications, such as coagulation disorders, anemia, and eclampsia; (2) multiple pregnancy; (3) Patients with critical obstetric conditions such as placental abruption and uterine rupture, as well as those requiring emergency cesarean delivery due to recurrent antenatal hemorrhage, uterine contractions and other indications; (4) Patients with FIGO Grade 3b or 3c PAS who underwent hysterectomy without attempted placental dissection (17); (5) incomplete data. The information collected included maternal age, pre-pregnancy BMI, gestational age at surgery, number of cesarean sections, number of miscarriages, history of intrauterine operations (abortion, diagnostic or therapeutic curettage, and other endoscopic procedures such as polypectomy, myomectomy, adhesiolysis, and foreign body removal), gestational diabetes mellitus, gestational hypertension, neonatal weight, ultrasound score, ultrasound signs, intraoperative blood loss，and FIGO staging. This study was approved by the Medical Ethics Committee of Maternal and Child Health Care Hospital of Shandong Province affiliated with Qingdao University (ethics number: NO. 2026-002). The flow diagram for patient selection is presented in Figure 1.

### 2.2 Ultrasonic image acquisition and interpretation

Image Acquisition：GE Voluson E8/E10 Doppler ultrasound machines with convex (1.0–5.0 MHz), endocavity (6.0–12.0 MHz) and linear probes (6.0–15.0 MHz) were adopted. Patients were scanned in supine position with moderately filled bladders. All scans were conducted by two senior obstetric sonographers with more than five years of dedicated PAS ultrasound experience. Standard static images and dynamic cine clips were stored following a unified scanning protocol. All patients underwent transabdominal scanning; transvaginal ultrasound was selectively used for cervical and lower uterine segment assessment with informed consent.

Image interpretation: All offline ultrasound assessments were independently completed by the two previously mentioned obstetric sonographers using a double-blinded evaluation protocol. Neither sonographer was informed of intraoperative blood loss or surgical results during separate scoring of all study cases. When disagreements existed regarding individual sonographic feature scores or total composite scores, the two operators jointly reviewed the images and discussed each indicator item by item according to standardized scoring criteria to establish a final consensus score.

### 2.3 Diagnostic and stratification criteria

#### 2.3.1 PAS ultrasonic scoring criteria

Ultrasound scoring criteria were based on the scoring system established by Chong et al. (Table 1).

**Table 1:** Placenta accreta scoring system.

Variables	0	1	2
Position of the placenta	Normal	Marginal placental previa or low lying placenta（Distance from the internal cervical os < 2 cm）	Completely placental previa
Thickness of the placenta	<3 cm	3–5 cm	>5 cm
Retroplacental clear zone	Continuity	Partial interruption	Disappeared
Bladder line	Continuity	Partial interruption	Disappeared
Placental lacuna	None	Present	Fused with boiling water sign^a^
Condition of the subplacental vascularity	Normal blood flow	The blood flow increased, forming a cluster	Bridging vessels at the placental basal plate
Blood sinus of cervix^b^	None	Present	Fused with boiling water sign
Morphology of cervix^c^	Complete	Incomplete	Disappeared
Number of prior cesarean delivery	0	1	≥2

a Boiling water sign: defined as numerous irregular, tortuous intraplacental vascular lacunae demonstrating turbulent low-resistance blood flow on color Doppler ultrasound.

b Blood sinus of cervix: defined as dilated anechoic vascular spaces within the cervical stroma beneath the placental edge.

c Morphology of cervix: Morphology is defined as incomplete when the local cervix exhibits thinning or irregularity. Morphology is considered to have disappeared when the cervical tissue is extensively infiltrated by placental villi, resulting in a blurred cervical structure.

#### 2.3.2 PAS clinical diagnostic criteria (based on FIGO consensus)

Intraoperative findings evaluated by senior obstetricians at cesarean delivery were defined as the standard for PAS diagnosis.

Grade 1 (placenta accreta): The uterus shows no obvious distension over the placental bed (placental “bulge”), no placental tissue is seen invading through the surface of the uterus, and there is no or minimal neovascularity. The placenta fails to separate spontaneously after routine obstetric maneuvers, requiring mechanical or surgical procedures.

Grade 2 (placenta increta): Bluish-purple discoloration, focal bulging and significant disordered hypervascularity are present over the placental bed. Placental tissue invades the myometrium without serosal penetration. Gentle cord traction causes uterine dimpling without placental separation.

Grade 3 (placenta percreta): Placental tissue breaches the uterine serosa. Lesions confined only to the uterine serosa are classified as Grade 3a; lesions invading the urinary bladder are Grade 3b; lesions invading the broad ligament, vaginal wall, pelvic sidewall or other pelvic tissues are defined as Grade 3c (with or without concurrent bladder involvement). Variable obliteration of the surgical plane between the uterus and adjacent organs can be observed in all Grade 3 subtypes (17).

All patients fulfilling Stage 1 or higher criteria were diagnosed with PAS.

#### 2.3.3 Intraoperative blood loss stratification criteria

Intraoperative hemorrhage ≥1,000 mL during cesarean delivery for PAS was defined as massive hemorrhage (18). Patients were divided into two subgroups: the massive hemorrhage group (blood loss ≥1,000 mL) and the non-massive hemorrhage group (0–999 mL). Intraoperative blood loss was quantified by measuring suction fluid volume and weighing blood-soaked hemostatic gauzes.

This binary stratification of blood loss was solely used for statistical predictive analysis of intraoperative massive hemorrhage.

### 2.4 PAS surgical procedures and delivery timing management

#### 2.4.1 Surgical procedures

All patients complicated by PAS in our center were managed by a dedicated multidisciplinary team following a predefined standardized stepwise haemostasis protocol. A fertility-sparing conservative approach with selective placental management was adopted for eligible patients. Loosely adherent placental tissue was carefully separated, whereas deeply implanted placental tissue without safe dissection plane was left *in situ* to avoid aggressive forced placental separation. For localized, well-demarcated implanted lesions, limited focal resection was performed followed by uterine reconstruction and haemostatic suturing. Routine surgical techniques included full-thickness continuous uterine sutures and horizontal mattress seromuscular reinforcement sutures to reduce incision oozing and postoperative pelvic adhesions. Bilateral ascending uterine artery ligation was conducted if primary haemostatic measures failed to control persistent bleeding from the placental bed. Combined cervical lifting sutures were applied for compressive haemostasis in patients with active haemorrhage originating from the lower uterine segment and cervix. Cases complicated by uterine rupture were excluded according to predefined criteria. This unified institutional surgical protocol minimized heterogeneity arising from inter-surgeon differences in operative and haemostatic strategies.

#### 2.4.2 Delivery timing management

The FIGO consensus (19) for PAS does not recommend a fixed gestational age for elective cesarean delivery. Adhering to the guideline's principle of multidisciplinary, risk-stratified individualized management, we developed a standardized institutional delivery protocol. For stable patients with suspected or confirmed PAS and no recurrent antepartum hemorrhage or fetal distress, elective cesarean delivery was scheduled at 34–37 weeks of gestation. The precise delivery timing was primarily determined by sonographic placental invasion characteristics, with prior multiple cesarean deliveries and other clinical risks as auxiliary references.

### 2.5 Statistical methods

Statistical analysis was performed using SPSS 30.0 software. Count data were expressed as frequencies (%). The chi-square test was used for intergroup comparisons of 2 × 2 fourfold tables and 3 × 2 contingency tables. Fisher's exact test was applied when the theoretical frequency was less than 5. Normally distributed measurement data were analyzed using the independent samples t test and presented as mean ± standard deviation (x ± s). Non-normally distributed data were compared using the Mann–Whitney U test and expressed as median (25th percentile, 75th percentile). All sonographic signs were stratified into three categories for baseline intergroup comparison. All sonographic markers were further dichotomized for subsequent univariate and multivariate logistic regression to identify independent risk factors for massive intraoperative hemorrhage. Receiver operating characteristic (ROC) curve analysis was performed to assess the diagnostic efficacy of ultrasound score in predicting cesarean intraoperative hemorrhage. The sensitivity, specificity, positive predictive value (PPV), negative predictive value (NPV), positive likelihood ratio (LR+), and negative likelihood ratio (LR−) of the scoring system for predicting hemorrhage were calculated. A *P* value < 0.05 was considered statistically significant.

## 3 Results

### 3.1 General information

**Figure 2:**
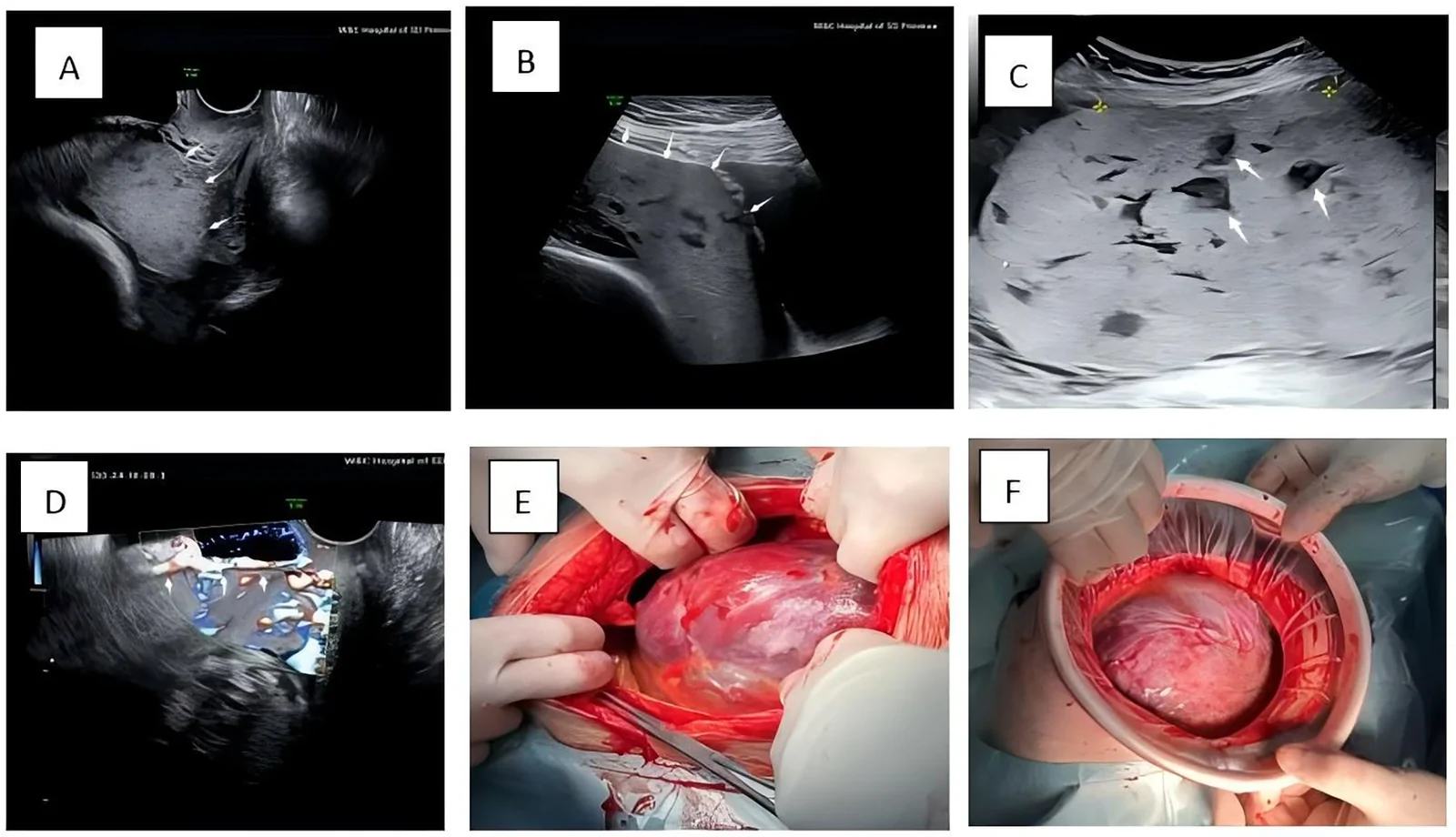
Typical ultrasound signs and intraoperative images. **(A)** Completely placental previa. **(B)** Disappearance of the retroplacental clear zone. **(C)** Placental lacunae. **(D)** Bridging vessels at the placental basal plate. **(E, F)** Intraoperative manifestations of placenta percreta. Placental tissue was observed to penetrate through the uterine serosa.

A total of 63 patients with PAS were enrolled, including 40 in the massive hemorrhage group and 23 in the non-massive hemorrhage group. Baseline comparisons of ultrasonic signs, sonographic scores, intraoperative blood loss and intraoperative FIGO grades between the two groups are shown in Table 2. Significant intergroup differences were observed in placental location, bladder interface morphology, basal placental vascularity, ultrasound score, intraoperative blood loss and FIGO grade (all *P* < 0.05). Representative ultrasonographic images are shown in Figure 2.

**Table 2:** Intergroup comparison of ultrasound signs, score, intraoperative blood loss and FIGO grade.

Variables	Massive hemorrhage group	Non-massive hemorrhage group	*χ*^2^/t	P
Position of the placenta			χ^2^ = 7.839	0.026（Fisher's exact test）
Normal	3（7.5%）	7（30.4%）		
Marginal placental previa or low lying placenta	5（12.5%）	5（21.8%）		
Completely placental previa	32（80.0%）	11（47.8%）		
Thickness of the placenta			χ^2^ = 4.326	0.113
< 3 cm	7（17.5%）	9（39.1%）		
3–5cm	19（47.5%）	10（43.5%）		
> 5 cm	14（35.0%）	4（17.4%）		
Retroplacental clear zone			χ^2^ = 4.879	0.083（Fisher's exact test）
Continuity	1（2.5%）	2（8.7%）		
Partial interruption	14（35.0%）	13（56.5%）		
Disappeared	25（62.5%）	8（34.8%）		
Bladder line			χ^2^ = 7.584	0.011（Fisher's exact test）
Continuity	26（65.0%）	22（95.7%）		
Partial interruption	13（32.5%）	1（4.3%）		
Disappeared	1（2.5%）	0（0.0%）		
Placental lacuna			χ^2^ = 5.536	0.069
None	4（10.0%）	5（21.8%）		
Present	20（50.0%）	15（65.2%）		
Fused with boiling water sign	16（40.0%）	3（13.0%）		
Condition of the subplacental vascularity			χ^2^ = 13.599	0.001
Normal blood flow	7（17.5%）	10（43.5%）		
The blood flow increased, forming a cluster	11（27.5%）	11（47.8%）		
Bridging vessels at the placental basal plate	22（55.0%）	2（8.7%）		
Blood sinus of cervix			χ^2^ = 3.237	0.225（Fisher's exact test）
None	30（75.0%）	21（91.3%）		
Present	6（15.0%）	2（8.7%）		
Fused with boiling water sign	4（10.0%）	0（0.0%）		
Morphology of cervix			χ^2^ = 4.409	0.114（Fisher's exact test）
Complete	30（75.0%）	22（95.7%）		
Incomplete	8（20.0%）	1（4.3%）		
Disappeared	2（5.0%）	0（0.0%）		
Ultrasound score	9.7 ± 2.8	6.0 ± 1.9	t = 5.607	0.001
Intraoperative blood loss	2,430 ± 1,134	660 ± 208	t = 43.443	0.001
FIGO grade			χ^2^ = 12.791	0.001（Fisher's exact test）
Grade 1	4（10.0%）	10（43.5%）		
Grade 2	27（67.5%）	13（56.5%）		
Grade 3	9（22.5%）	0（0.0%）		

### 3.2 Analysis of the predictive value of the ultrasound score for massive intraoperative hemorrhage during cesarean section in PAS patients

#### 3.2.1 Univariate analysis of risk factors for intraoperative massive hemorrhage in PAS patients

Univariate analysis was performed to identify risk factors for intraoperative massive hemorrhage (Table 3). The hemorrhage group delivered significantly earlier, presented more frequent histories of ≥2 prior cesarean deliveries, and achieved much higher sonographic scores (9.7 ± 2.8 vs. 6.0 ± 1.9, *P* < 0.001). No significant intergroup differences were observed in other clinical baseline variables (all *P* > 0.05).

**Table 3:** Univariable analysis of massive hemorrhage during cesarean section in PAS patients.

Variables	Massive hemorrhage group	Non-massive hemorrhage group	χ^2^/t/z	P
Maternal age (years)	34.2 ± 2.9	33.2 ± 4.2	t = 1.224	0.226
Gestational age at surgery (weeks)	35.4 ± 1.4	36.8 ± 1.3	t = - 4.104	0.001
Pre-pregnancy BMI (kg/m^2^)	23.5 (22.2,26.2)	22.5 (20.5,24.3)	z = 1.606	0.108
Number of cesarean sections (number)				0.019（Fisher's exact test）
0	2（5.0%）	4（17.4%）		
1	16（40.0%）	14（60.9%）		
≥2	22（55.0%）	5（21.7%）		
Number of abortions			χ^2^ = 0.292	0.864
0	12 (30.0%)	6 (26.1%)		
1	15 (37.5%)	8 (34.8%)		
≥2	13 (32.5%)	9 (39.1%)		
History of intrauterine operations			χ^2^ = 1.370	0.242
Yes	20 (50.0%)	15 (65.2%)		
No	20 (50.0%)	8 (34.8%)		
Gestational hypertension				0.753（Fisher's exact test）
Yes	9 (22.5%)	4 (17.4%)		
No	31 (77.5%)	19 (82.6)		
Gestational diabetes mellitus			χ^2^ = 2.315	0.128
Yes	12 (30.0%)	3 (13.0%)		
No	28 (70.0%)	20 (87.0%)		
Birth weight (g)	2,700 ± 472	2,769 ± 481	t = −0.552	0.583
Ultrasound score	9.7 ± 2.8	6.0 ± 1.9	t = 5.607	0.001

For more concise univariate statistical analysis and better clinical applicability, the three-tier sonographic signs were dichotomized, and the related univariate results are shown in Table 4. Table 4 shows six sonographic signs correlated with intraoperative massive hemorrhage (all *P* < 0.05): complete placenta previa, disappearance of the retroplacental clear zone, partial interruption or disappearance of the bladder line, placental lacunar fusion with the boiling water sign, bridging vessels at the placental basal plate, and abnormal cervical morphology. Cervical sinus was more prevalent in the hemorrhage group without statistical significance (*P* = 0.183).

**Table 4:** Univariable analysis of ultrasound signs of massive hemorrhage during cesarean section in PAS patients.

Variables	Massive hemorrhage group	Non-massive hemorrhage group	χ^2^	P
Complete placenta previa			6.977	0.008
Yes	32 (80.0%)	11 (47.8%)		
No	8 (20.0%)	12 (52.2%)		
Placental thickness ≥ 5 cm			2.219	0.136
Yes	14 (35.0%)	4 (17.4%)		
No	26 (65.0%)	19 (82.6%)		
Disappearance of the retroplacental clear zone			4.498	0.034
Yes	25 (62.5%)	8 (34.8%)		
No	15 (37.5%)	15 (65.2%)		
Partial interruption or disappearance of the bladder line			7.563	0.006
Yes	14 (35.0%)	1 (4.3%)		
No	26 (65.0%)	22 (95.7%)		
Placental lacunar fusion with the boiling water sign			5.038	0.025
Yes	16 (40.0%)	3 (13.0%)		
No	24 (60.0%)	20 (87.0%)		
Bridging vessels at the placental basal plate			13.277	0.001
Yes	22 (55.0%)	2 (8.7%)		
No	18 (45.0%)	21 (91.3%)		
Cervical sinus				0.183（Fisher's exact test）
Yes	10 (25.0%)	2 (8.7%)		
No	30 (75.0%)	21 (91.3%)		
Abnormal cervical morphology				0.044（Fisher's exact test）
Yes	10 (25.0%)	1 (4.3%)		
No	30(75.0%)	22(95.7%)		

#### 3.2.2 Independent risk factors for intraoperative massive hemorrhage: multivariate logistic regression analysis

Multivariate logistic regression was performed to adjust confounders from variables significant on univariate analysis. As shown in Table 5, the sonographic score was an independent risk factor for intraoperative massive hemorrhage (OR = 1.774, 95% CI: 1.192–2.640, *P* = 0.005).

**Table 5:** Multivariable logistic analysis of massive hemorrhage during cesarean section in PAS patients.

Variables	β	SE	Wald	*p* value	OR	95% CI	
Gestational age at surgery (weeks)	−0.461	0.322	2.024	0.153	0.631	0.335	1.187
Number of cesarean sections (number)	0.539	0.500	1.159	0.282	1.714	0.643	4.567
Ultrasound score	0.573	0.203	7.996	0.005	1.774	1.192	2.640

Separate multivariate regression for individual sonographic signs (Table 6) identified complete placenta previa and bridging vessels at the placental basal plate as independent predictors of intraoperative massive hemorrhage: complete placenta previa (OR = 10.199, 95% CI: 1.794–57.977, *P* = 0.009), bridging vessels at the placental basal plate (OR = 15.750, 95% CI: 1.591–155.867, *P* = 0.018).

**Table 6:** Multivariable logistic analysis of ultrasound signs of massive hemorrhage during cesarean section in PAS patients.

Variables	β	SE	Wald	*p* value	OR	95% CI	
Complete placenta previa	2.322	0.887	6.86	0.009	10.199	1.794	57.977
Disappearance of the retroplacental clear zone	0.130	0.693	0.035	0.851	1.139	0.293	4.434
Partial interruption or disappearance of the bladder line	1.580	1.377	1.317	0.251	4.856	0.327	72.186
Placental lacunar fusion with the boiling water sign	0.096	1.078	0.008	0.929	1.101	0.133	9.113
Bridging vessels at the placental basal plate	2.757	1.170	5.557	0.018	15.750	1.591	155.867
Abnormal cervical morphology	0.454	1.374	0.109	0.741	1.575	0.107	23.251

#### 3.2.3 Predictive performance of the sonographic scoring system for intraoperative massive hemorrhage

**Figure 3:**
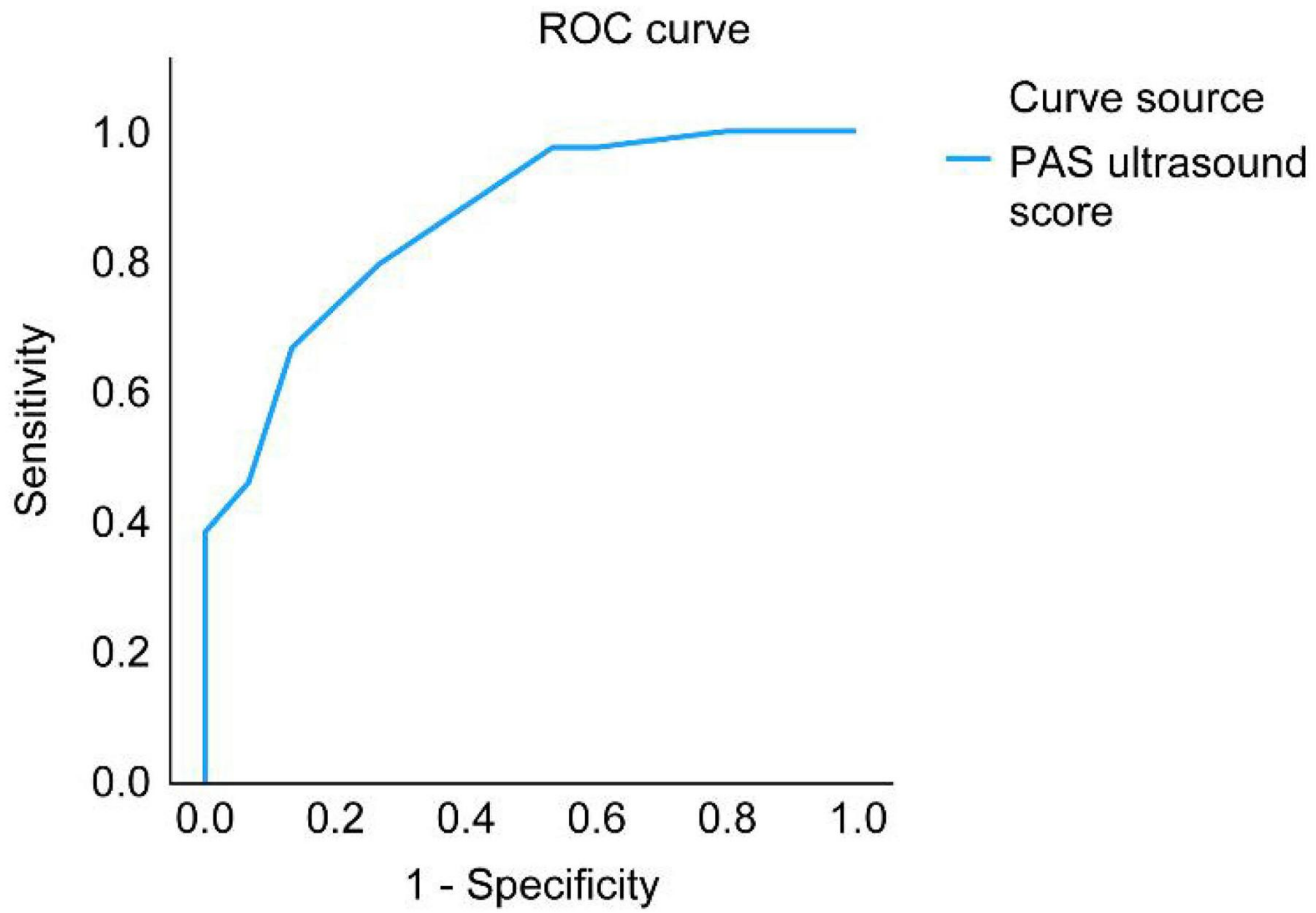
ROC curve for using ultrasound score to predict intraoperative hemorrhage during cesarean section in PAS patients.

ROC curve analysis was performed to evaluate the overall predictive performance of the sonographic score for intraoperative massive hemorrhage. As shown in Figure 3, the AUC was 0.884 (95% CI: 0.804–0.963, *P* < 0.01), showing good discriminative ability. At the optimal cut-off of 7.5 points, the score achieved a sensitivity of 80.0%, specificity of 82.6%, PPV of 88.9%, NPV of 70.4%, LR + of 4.598, and LR– of 0.242.

## 4 Discussion

Prenatal ultrasound serves as the primary imaging modality for screening and risk stratification of PAS. A multitude of standardized sonographic grading and quantitative scoring tools have been developed worldwide to facilitate PAS diagnosis and severity stratification (11, 20–23). Clinically, cervical placental invasion represents a major contributor to catastrophic intraoperative hemorrhage during cesarean delivery. The Chong scoring system uniquely incorporates standardized metrics for cervical involvement, enabling comprehensive evaluation of hemorrhage risks stemming from placental invasion of the lower uterine segment and cervix. For this reason, the present study utilized this scoring framework to conduct preoperative bleeding risk assessment.

Analysis of sonographic features based on the Chong scoring system demonstrated that patients in the massive hemorrhage group more frequently presented with complete placenta previa, abnormal bladder line and bridging vessels at the placental basal plate. These findings were consistent with previous studies reporting that sonographic signs including disappearance of the retroplacental clear zone and abnormal bridging vessels possess high diagnostic value (24, 25). Further comparisons of baseline data revealed that the hemorrhage group had significantly higher sonographic scores, greater intraoperative blood loss and more severe FIGO grades, indicating that the depth of placental invasion and severity of vascular abnormalities are closely correlated with intraoperative bleeding risk.

To eliminate confounding factors and clarify the independent predictive effect of the Chong scoring system, all clinical and ultrasonic variables in this cohort were included for multivariate regression analysis. All cesarean deliveries and hemostatic procedures were performed by a specialized multidisciplinary team using standardized surgical protocols, which completely eliminated operative bias derived from inter-operator variability. After adjustment, the preoperative Chong score was identified as an independent risk factor for intraoperative massive hemorrhage in our elective PAS population. Unlike a previous study (26) that enrolled heterogeneous cases including emergency surgery and anemic patients, the present study strictly included only elective and non-anemic patients, further verifying the stable and independent predictive performance of the Chong scoring system in standardized PAS cohorts. The scoring system can objectively reflect the invasive depth of the placenta into the myometrium and bladder as well as the severity of vascular malformations, thereby facilitating precise preoperative risk stratification and individualized surgical decision-making for elective PAS patients (27).

ROC curve analysis was further conducted to verify the preoperative predictive efficacy of the Chong scoring system. At the optimal cut-off value of 7.5 points, the AUC reached 0.884 with favorable sensitivity and specificity. The high positive predictive value implied that patients with scores ≥7.5 points carry a sharply elevated risk of intraoperative massive hemorrhage, requiring comprehensive preoperative blood preparation and activation of multidisciplinary surgical protocols. The low negative likelihood ratio indicated that patients with scores below 7.5 points have a low probability of severe bleeding, which avoids excessive consumption of medical resources. The cut-off threshold can serve as an objective standardized tool for preoperative risk stratification of PAS, assist clinical risk communication and allocation of perioperative resources, and ultimately improve the overall safety of PAS-related surgeries.

Although the total score can effectively evaluate overall bleeding risk, patients with identical scores still display individual differences in intraoperative blood loss. Thus, separate analysis of the independent predictive value of each individual sonographic sign provides critical supplementary evidence. Prior studies have proven that complete placenta previa, abnormal basal placental vessels, cervical vascular lacunae, loss of the retroplacental clear zone and disrupted bladder line are closely associated with adverse PAS outcomes (28–30). After dichotomous transformation of all ultrasonic signs in our study, complete placenta previa and bridging vessels at the placental basal plate were identified as two independent risk factors for massive intraoperative hemorrhage. The underlying pathological mechanisms are as follows: complete placenta previa attaches to the poorly contractile lower uterine segment and cervix, resulting in insufficient wound contraction and impaired hemostasis after placental separation. Bridging vessels at the placental basal plate indicate deep trophoblastic invasion of the myometrium accompanied by aberrant spiral artery remodeling and vascular vasoconstrictive dysfunction. These two pathological alterations synergistically increase the risk of refractory massive hemorrhage (31, 32). Notably, although disappearance of the retroplacental clear zone and abnormal bladder line differed significantly between groups in univariate analysis, they lost independent predictive value after confounding adjustment, which was mainly attributed to severe collinearity. Both signs reflect a continuous pathological progression of placental invasion toward the serosa and bladder with substantial overlapping information. This finding suggests that these correlated sonographic manifestations can be integrated into composite indicators in future research to more accurately evaluate the risk of deep placental invasion. In addition, blood sinus of cervix and abnormal cervical morphology were more prevalent in the hemorrhage group, but failed to demonstrate independent predictive significance due to the limited number of positive cases.

In summary, a preoperative sonographic score of 7.5 points can be used as a reliable cut-off value to predict massive intraoperative hemorrhage in PAS patients. For high-risk patients with scores ≥7.5 points complicated by complete placenta previa and bridging vessels at the placental basal plate, the risk of severe intraoperative bleeding is further elevated. Clinicians should maintain high vigilance for such patients, implement thorough high-level preoperative preparation and multidisciplinary interventions, so as to optimize maternal and neonatal prognoses.

In addition, the Chong scoring system adopted in this study can effectively differentiate true PAS and non-PAS uterine scar dehiscence. Non-PAS scar defect merely presents focal myometrial thinning with intact placental morphology and continuous retroplacental interface. In contrast, PAS cases exhibit typical invasive sonographic features caused by villous infiltration and abnormal vascular remodeling. This etiology-based discrimination excludes confounding bleeding from simple scar dehiscence and further validates the reliability of our blood loss stratification results.

Nevertheless, the Chong scoring system still has inherent clinical limitations despite its convenience and rapidity in preoperative risk stratification. Mismatches between ultrasound scores and actual intraoperative severity were observed in several cases of this study. Deep placental invasion and subtle cervical infiltration tend to be underestimated by transabdominal ultrasound due to limited resolution and ambiguous placental-myometrial interfaces, resulting in lower scores and underestimated bleeding risk. Conversely, pelvic varicose veins and imaging artifacts may be misidentified as abnormal bridging vessels and disrupted bladder line, leading to overestimated scores. Furthermore, some patients cannot tolerate transvaginal ultrasound, which restricts the precise assessment of cervical involvement and constitutes an unavoidable limitation of this scoring system.

This study has several limitations. First, it was a single-center retrospective analysis without *a priori* sample size estimation. The relatively small sample size might have limited the statistical power to detect weak predictive effects of infrequent sonographic signs, and further large-scale, multicenter prospective studies are warranted to validate our findings. Second, several ultrasonic signs involved in the Chong scoring system, including fused placental lacunae (boiling-water sign), cervical sinus, and abnormal cervical morphology, have not been adequately validated in previous PAS studies. Further multicenter prospective studies are required to confirm the diagnostic value and generalizability of these understudied ultrasonic indicators. Third, the lack of a unified global criterion for PAS-related massive hemorrhage means our threshold of intraoperative blood loss ≥1,000 mL may restrict the generalizability of the results. Fourth, only patients undergoing elective cesarean delivery were included. The predictive value of the Chong scoring system for early-gestation PAS and emergency cesarean cases remains unclear and requires further prospective verification.

## 5 Conclusions

PAS is an extremely dangerous obstetric complication. Intraoperative hemorrhage may directly lead to hysterectomy or even maternal death. Preoperative identification of risk factors related to PAS and determination of the cutoff value of the ultrasound score are of great significance for formulating surgical plans and improving patient outcomes.

## Data Availability

The datasets used and/or analysed during the current study are available from the corresponding author on reasonable request. Access to individual patient-level data is restricted due to ethical requirements and patient privacy protection.
